# Prevalence of depression among people with type 2 diabetes mellitus: a cross sectional study in Palestine

**DOI:** 10.1186/1471-2458-14-163

**Published:** 2014-02-13

**Authors:** Waleed M Sweileh, Hanadi M Abu-Hadeed, Samah W Al-Jabi, Sa’ed H Zyoud

**Affiliations:** 1 Department of Pharmacology/ Toxicology, College of Medicine and Health Sciences, An-Najah National University, Nablus, Palestine; 2 Qalqilia Governmental Hospital, Ministry of Health, Qalqilia, Palestine; 3 Department of Clinical and Comunity Pharmacy, College of Medicine and Health Sciences, An-Najah National University, Nablus, Palestine

**Keywords:** Diabetes mellitus, Depression, Palestine

## Abstract

**Background:**

Diabetes mellitus is a common chronic metabolic disorder and one of the main causes of death in Palestine. Palestinians are continuously living under stressful economic and military conditions which make them psychologically vulnerable. The purpose of this study was to investigate the prevalence of depression among type II diabetic patients and to examine the relationship between depression and socio-demographic factors, clinical factors, and glycemic control.

**Methods:**

This was a cross-sectional study at Al-Makhfiah primary healthcare center, Nablus, Palestine. Two hundred and ninety-four patients were surveyed for the presence of depressive symptoms using Beck Depression Inventory (BDI-II) scale. Patients' records were reviewed to obtain data pertaining to age, sex, marital status, Body Mass Index (BMI), level of education, smoking status, duration of diabetes mellitus, glycemic control using HbA1C test, use of insulin, and presence of additional illnesses. Patients’ medication adherence was assessed using the 8-item Morisky Medication Adherence Scale (MMAS-8).

**Results:**

One hundred and sixty four patients (55.8%) of the total sample were females and 216 (73.5%) were < 65 years old. One hundred and twenty patients (40.2%) scored ≥ 16 on BDI-II scale. Statistical significant association was found between high BDI-II score (≥ 16) and female gender, low educational level, having no current job, having multiple additional illnesses, low medication adherence and obesity (BMI ≥ 30 kg/m2). No significant association between BDI score and glycemic control, duration of diabetes, and other socio-demographic factors was found. Multivatriate analysis showed that low educational level, having no current job, having multiple additional illnesses and low medication adherence were significantly associated with high BDI-II scores.

**Conclusion:**

Prevalence of depression found in our study was higher than that reported in other countries. Although 40% of the screened patients were potential cases of depression, none were being treated with anti-depressants. Psychosocial assessment should be part of routine clinical evaluation of these patients at primary healthcare clinics to improve quality of life and decrease adverse outcomes among diabetic patients.

## Background

Diabetes mellitus (DM) is a common health problem with serious medical and economic consequences. Between 2010 and 2030, there will be a 69% increase in numbers of adults with diabetes in developing countries and a 20% increase in developed countries [1]. The Arab world (North Africa, Middle East, and Gulf area) will have second highest increase in percentage of people with DM in 2030 compared to other parts of the world [1]. Few studies about prevalence of DM were carried out in Palestine and showed a higher rate of this disease in an urban community than in a rural community [2-4]. However, no reliable data exist for treatment, complications, economic effect, and outcomes of treatment of DM in Palestine [5].

Depression is another prevalent condition. Approximately 340 million people worldwide suffer from depression at any given time [6]. A study designed to examine the prevalence of mood disorders in 14 countries found that the 12-month prevalence of mood disorders was lowest in Nigeria and highest in the United States [7]. It was estimated that depressive disorders were the fourth leading cause of disease burden in women and seventh leading cause in men [8,9]. Major depression was found to be the second leading cause of disability-adjusted life years (DALYs) lost in women and the tenth leading cause of DALYs in men [10]. In Palestine, psychological distress is high, quality of life is very low, and daily life of Palestinians is constantly under threat which make Palestinians more vulnerable to stress and depression [5].

Relationship between DM and depression has been investigated by many researchers. Prevalence of depression among individuals with DM appears to vary by type of DM, race/ethnicity, and among developed and developing nations [11,12]. Therefore, screening for depression among diabetic patients is important in different races and ethnicities. Actually, studies indicated that about 49% of the diabetic patients having severe depression were misrecognized on the primary healthcare clinics [13,14]. Unfortunately, Palestinians with chronic diseases are usually less likely to have regular sources of medical care for screening or preventive services [15].

The aim of this study was to estimate the prevalence of depression among Palestinian type 2 diabetic patients attending primary healthcare settings and to describe socio-demographic and clinical characteristics associated with diabetic patients having depression. Given the scarcity of research about depression among Palestinians in general and among diabetic patients in particular, such study is needed. The knowledge gained from this study will assist healthcare practitioners to better understand depression in diabetes mellitus and design treatments that address the psychological and the metabolic needs of affected individuals to improve overall health outcomes.

## Methods

### Setting and design

This was a cross-sectional descriptive study for the purpose of evaluating the presence of depression symptoms and their association with glycemic control (HbA1C), medication adherence, clinical and demographic variables among Palestinians with type 2 diabetes mellitus.

Nablus is the largest city in north West-Bank of Palestine. Residents of Nablus city are predominantly Arabs. The study was carried out at the governmental diabetes primary healthcare clinic (Al-Makhfia) in Nablus city. There are five main healthcare providers of health services in Palestine: Ministry of health, UNRWA, NGOs, Palestinian Military Medical Services (PMMS) and Private for profit. MOH bears the heaviest burden, as it has the major responsibility [16]. During the study period, the investigator visited the primary healthcare center at Al-Makhfia, Nablus. The visits were made on Sundays, Tuesdays, Wednesdays and Thursdays every week. These are the assigned days for diabetic clinics to deliver care for patients with type II DM. Al-Makhfia center is the only governmental center that provides care for diabetic patients with governmental insurance in Nablus city. The study was carried out from April to August 2012.

### Sample population

This study included a convenience sample of adult population. Participants were recruited from Al-Makhfia diabetic clinics while waiting to be seen by their health care providers. The inclusion criteria for this study were: 1) males and females 18 years old and older who self-report being diagnosed with type 2 diabetes by a health care provider and has a medical file at the diabetic clinic; 2) self-report type 2 diabetes for one year or longer; 3) under medical care for diabetes treatment according to MOH documents; 4) able to understand the questions in order to help complete forms and questionnaires; 5) willingness to participate in this study and finally 6) being scheduled to do HbA1c at the laboratory of MOH at the time of the visit. The exclusion criteria were based on self-report of the following: 1) physical and or mental conditions that interfere with participation, and 2) inability to obtain venous blood sample.

### Sample size

No studies about the prevalence of depression among diabetic patients in Palestine have been reported. Therefore, we estimated the sample size based on studies in other Arab countries [17]. The sample size was estimated based on the following assumptions: a descriptive study with dichotomous outcome: the sample size tables shows that a sample of 246 participants is needed if we assume the prevalence of depression to be 20% and the width of confidence interval to be 10% and the confidence limit to be 95%.

### Participant recruitment procedure

The investigators obtained written approval from MOH to carry out this study. A brief screening for recruitment of participants was conducted by the investigators to identify potential participants in the following manner: every person in the waiting area was asked if he is willing to talk to the investigator. If the person agreed to talk to the investigator for possible participation, the attending specialist was asked if that person is scheduled to do HbA1c at that visit. If the person who agreed was scheduled to do HbA1c, then an informed consent was read and obtained by the investigators at the diabetic clinic. Once the consent was signed, verification of inclusion and exclusion criteria took place. The questionnaires required for the study were presented and explained for its completion during this session. The forms and questionnaires that were completed included: 1) demographic and clinical questionnaire to gain information on participant, 2) BDI-II to measure depressive symptoms), Morisky Medication Adherence Scale (MMAS-8) to determine level of medication adherence among participants.

An investigator, who is a graduate nursing student with a long experience in nursing, was trained prior to initiation of the study to assist in the recruitment and administration of questionnaires. Re-training of the investigator who administered the questionnaire took place after the first week of the research study based on the need to clarify certain points in the questionnaire. All participants completed the questionnaires in a private area in the clinic. A venous blood test, in a non-fasting state, was drawn for measuring the HbA1C during the same session. A certified phlebotomist was available on site to obtain the sample and forward it to official labs of the MOH. Results regarding HbA1c for each participant were available to the PI within the same working day. Data collection and the completion of questionnaires took place before HbA1c test. A note was given to healthcare provider in the clinic about each participant’s HbA1c results.

### Instruments

#### Measure of depression symptoms

The presence of depression symptoms was evaluated using the Beck Depression Inventory-Second Edition (BDI-II) [18]. Beck Depression Inventory is a well-known self-report instrument. Its original version (21 items) was introduced in 1961 and its reliability and validity have been established across a broad spectrum of clinical and non-clinical populations [19-25]. The BDI-II is available in English and has been translated into Arabic and validated for use to measure depression. The BDI-II has been translated into Arabic and validated for use to measure depression. Use Permission to use the Arabic version of BDI-II was obtained from the author who did the translation and validation [26,27].

The term “depression” in this study referred to the self-report of depressive symptoms identified in the BDI-II. The format of the BDI-II test is for the participants to select and circle the number beside one of the four phrases listed that best describes their state in the past two weeks including the day the questionnaire is answered. The instrument consists of 21-items/statements that are self-reported and takes approximately 15 minutes to complete. The score ranges from 0 - 63 to determine possible degree of depression symptoms. The instrument developers established four groups of scores and classified as the following: “minimal 0-13, mild 14-19, moderate 20-28, and severe 29-63” [18]. Scores provide an estimate of the overall symptom severity of depression. High scores indicate greater depressive symptoms. We used the most commonly used cut-off scores for BDI of ≥ 16 to indicate clinical depression [28]. It should be emphasized that high BDI II scores presented in this study are not definitive diagnosis of depression. Rather, high scores are indicative of the presence of depressive symptoms and further clinical consultation to establish definitive diagnosis of depression is needed.

#### Serum glycated hemoglobin measure (HbA1C)

Glycated hemoglobin (HbA1c) is used to measure blood glucose control over several months and provides an estimate of how well diabetes has been controlled over the last 2 or 3 months. It is the goal standard of care for determining potential risk for developing problems, such as retinopathy, renal disease, cardiovascular disease, peripheral neuropathy or stroke. Potential complications are especially true if HbA1C remains high for a long period of time [29]. The goal for HbA1C in adults is < 7% [29]. Serum blood test for HbA1C was obtained on the same day and in the same clinic site either before or immediately after completing the questionnaires depending on time available in clinic. The blood sample was obtained by venous puncture drawn by a certified phlebotomist and sent to a designated local laboratory with coded identification number. HbA1c determination for all samples was done in the same laboratory by the same technician and applying the same technique. The method used to determine HbA1c is NycoCard READER II, using the Boronate affinity test principle. It is NGSP-certified method.

#### Medication adherence

Anti-diabetic Medication adherence was measured using Morisky Medication Adherence Scale (MMAS-8) [30]. Approval to use and translate the MMAS-8 into Arabic language was obtained from the developer. The translation was carried out according to standard forward and backward method. The Arabic- translated version of MMAS-8 was used in previous publication [31]. The MMAS-8 is an eight items questionnaire designed to measure medication adherence. It is composed of seven Yes/No questions. Details of the Arabic translation and its use were described previously [31]. The eighth question is a 5-points likert scale. In this study, patients with a total score of MMAS-8 less than 6 are considered to have poor medication adherence.

#### Demographic and health questionnaire

A questionnaire was designed for this study to gain information that would assist in obtaining biographical and health history of the participants. The contents included personal descriptive data such as age, gender, years of education, and income. Personal identification information that may breach confidentiality of participants was not be included. The inclusion of address was obtained separately to be able to send results of HbA1C, if desired. Information was obtained on medical history, current pharmacological diabetes treatment, and whether treatment for depression was in progress. The medical history components is a list of nine illnesses commonly identified with type 2 diabetes where the response is in a dichotomous (yes/no) format with an option to write in additional illnesses not included in the list. All the participants were asked to report all the medications that they use on chronic basis. For the purpose of this manuscript, presence of ischemic heart disease and/or congestive heart failure and/or arrhythmia are referred to collectively as cardiac problems while the presence of any stage of chronic kidney disease is referred to as renal problems. The data reported by the participants regarding their medications was validated through checking the computerized system at the MOH which contained up-to-date information about the patients and their medications. The questionnaire was available in Arabic language.

### Data management and statistical analysis

#### Pre-analysis

During the pre-analysis phase, the data were coded to maintain confidentiality for all participants. Participants were given an identification number assigned by the PI for use throughout the study. In order to carry out quantitative statistical analysis, measurement for variables was established. Demographic information and summative score of the instruments was obtained as a continuous measure as much as possible to ease the process of designation measure according to statistical analysis. The data were entered onto an SPSS (Statistical Package for the Social Sciences) statistical software was used for data analysis.

#### Statistical analyses

Descriptive statistics was carried out for all variables and expressed as mean ± SD for continuous variables with normal distribution. Non-normally distributed continuous variables were expressed as median (Q1-Q3). Normality of the data was tested by Kolomogrov-Smirnov test. Factors associated with depression scores > 16 were analyzed with binary logistic regression followed by multiple logistic regression analysis. Variables that showed significance in univariate analysis were included in multiple logistic regression analysis. The dependent variable was depression scores (≥ 16 versus < 16). A p value of < 0.05 was considered statistically significant.

#### Protection of human subjects

The study was undertaken as part of a higher research degree at An-Najah National University, Nablus, Palestine. The study protocol was approved by the ethics committee called “Institutional Review Board” (IRB) of the college of Medicine and Health Sciences, Palestine. The application for ethics committee was submitted on May 01, 2012 and the approval was obtained on June 04, 2012. The interviewer explained the purpose of the study and procedures and gained written informed consent before commencing the interview. The participants were also informed that their participation was voluntary and that they could withdraw from the interview/study at any time without consequences. The participants were also informed about potential risks for participating in the study which were few and included any of the following: (1) becoming fatigued or nervous while completing the questionnaires, (2) possibility of pain to site during needle stick, (3) a hematoma at the venous puncture site, and/or (4) infection, although it is highly unlikely, from the needle stick. The participants were assured that their responses would be treated in confidence and they were assured anonymity through the use of strict coding measures. All information was kept confidential and consent forms were number coded for identification. The research file will be kept for five years after completion of the study. At the end of this period, records will be destroyed appropriately. The study was carried out in full compliance with the guidelines of good clinical practice of the world assembly declaration of Helsinki and was approved by the university ethical committee. Well informed consent was taken from all enrolled patients.

## Results

During the study period, approximately 1400 patients visited the center and did the HbA1c test per required by the physician. Many of those patients leave the clinic immediately after taking the blood sample which made them un-available for interview. A net total of 301 diabetic patients were available and agreed to do the interview. Seven patients were excluded because of their inability to communicate or understand the questions or have sensory impairment like being deaf. A net of 294 type 2 diabetic patients met the inclusion criteria and were interviewed and their HbA1c test was obtained.

The majority (216; 73.5%) of participants were younger than 65 years old. Age was negatively skewed with a median (Q1 – Q3) age of 60 (52 – 66.25) years. The majority (164; 55.8%) of participants was female. The largest portion (n = 213, 72.5%) of participants reported being either illiterate or had a limited school education while those with college education were minority (n = 40; 13.6%). The marital status as reported by participants was married (n = 243; 82.7%), single/divorced/widowed (n = 51, 17.3%). A small proportion (n = 62, 21.1%) of the participants reported that they have a current job. The majority (n = 223, 75.9%) of participants were non-smokers at the time of the study. Participants reported the number of years since diagnosis with type 2 diabetes which showed a wide distribution ranging from 5 – 35 years, positive skewness with a median (Q1 – Q3) of 10 (5 – 16) years. The Body Mass Index (BMI) of the participants showed positive skewness with a median (Q1 – Q3) of 30.6 (27.2 – 35.1). The BMI for males was 28.8 (26.6 – 33.8) while that for females was 32 (28.3 – 37.4). The difference in BMI between males and females was significant (P < 0.01) with a Z score of – 3.44. More than half (166; 56.5 %) of the participants had a BMI ≥ 30. Both systolic and diastolic blood pressures (SBP, DBP) showed positive skewness. The median (Q1 – Q3) values for SBP and DBP were 132 (120 – 143) and 80 (75 – 88) mmHg respectively.

The number of diabetes medications reported by participants ranged from 0-3 types with a median (Q1 – Q3) of 2 (1 – 2) medications. One hundred and forty seven (50%) reported insulin use either as a monotherapy or in combination with other medications for diabetes management. Two hundred and sixty participants acknowledged having additional illnesses along with diabetes. The number of additional illnesses ranged from 1 – 5 with a median (Q1 – Q3) of 2 (1 - 3). The most common chronic illness reported by the participants was high cholesterol (n = 219, 74.5%), followed by hypertension (n = 178, 60.5%), cardiac problems (n = 84, 28.6%) and renal problems (37, 12.6%). In the reported medical history, none of the participants recalled being informed that they had depression. Additionally, none of the participants reported taking medications for depression.

The results for HbA1C ranged from 5.2 to 13%, a median (Q1 – Q3) of 8.2% (7.2 – 9.1). The median and mean scores of HbA1C exceed the recommendation of < 7.0% established by the ADA [29]. The results were un-equally distributed with 3 extreme scores representing HbA1C between 12 and 13%. The HbA1C results were further divided into two categories: controlled (< 7%) and uncontrolled (≥ 7.0%). The percentage of participants who had a controlled glucose was 17.7% (n = 52). However, 82.3% (n = 242) were in the uncontrolled level which places individuals at a greater risk for developing diabetic complication.

The depression symptoms score distribution was positively skewed with the majority of participants [n = 174, 59.2%] scored less than 16 while 120 (40.2%) patients scored ≥ 16 in the depression scale. Medication adherence scores were negatively skewed. The median value for medication adherence scores was 5.8 (4.8 – 6.8). The majority (n = 167, 56.8%) of the participants had low adherence scores (MMAS-8 < 6). Table 1 shows univariate analysis of demographic and clinical factors with depression scores. The analysis showed that female diabetic patients, low level of education, having no current job, having higher number of additional illness, low medication adherence score and having high BMI were significantly associated with depression score of ≥ 16. Multivariate analysis (Table 2) showed that: 1) diabetic patients with college education were less likely to have depression [O.R = 0.24; (0.09 – 0.66)] than those with lesser level of education, 2) diabetic patients with no current job were more likely to have depression [O.R = 2.78 (1.23 – 6.27)] than those who are currently working, 3) diabetic patients with multiple additional illnesses were more likely to have depression [O.R = 1.81 (1.05 – 3.11)] than those with ≤ 1 additional illness, and finally 4) diabetic patients with high medication adherence score were less likely to have depression [O.R = 0.31 (0.18 – 0.53)] than those with low (< 6) medication adherence score.

**Table 1:** Univariate analysis of factors associated with depression

**Variable**	**Total**	**BDI-II depression score**		**Unadjusted OR**	**P-value**
	**N = 294**				
				**(95% CI)**	
		**≥ 16**	**< 16**		
		**N = 120**	**N = 174**		
**Age (years)**					
≤ 65	216 (73.5%)	85 (39.4%)	131 (60.6%)	0.8 (0.5 – 1.3)	0.40
> 65	78 (26.5%)	35 (44.9%)	43 (55.1%)	Reference	
**Gender**					
Male	130 (44.2%)	43 (33.1%)	87 (66.9%)	Reference	0.02
Female	164 (55.8%)	77 (47.0%)	87 (53.0%)	1.8 (1.1 – 2.9)	
**Education**					
Illiterate	87 (29.6%)	47 (39.2%)	40 (23.0%)	Reference	
Elementary	126 (42.9%)	53 (44.2%)	73 (42.0%)	0.6 (0.4 – 1.1)	0.08
High school	41 (13.9%)	13 (10.80%)	28 (16.1%)	0.4 (0.2 – 0.9)	0.02
College	40 (13.6%)	7 (5.8%)	33 (19.0%)	0.2 (0.1 – 0.5)	< 0.01
**Marital status**					
Married	243 (82.7%)	101 (41.6%)	142 (58.4%)	1.2 (0.6 – 2.2)	0.58
Others	51 (17.3%)	19 (37.3%)	32 (62.7%)	Reference	
**Currently smoking**					
Yes	71 (24.1%)	32 (45.1%)	39 (54.9%)	1.3 (0.7 – 2.2)	0.40
No	166 (56.5%)	79 (47.6%)	84 (52.4%)	Reference	
**Working**					
Yes	62 (21.1%)	12 (19.4%)	50 (80.6%)	Reference	< 0.01
No	232 (78.9%)	108 (46.6%)	124 (53.4%)	3.6 (1.8 – 7.7)	
**Duration of illness**	10 (5 – 16)	12 (6 – 16.75)	10 (5 – 15)	1.0 (0.9 – 1.05)	0.32
**Number of additional illness**					
≤ 1	114 (38.8%)	36 (31.6%)	78 (68.4%)	Reference	0.01
≥ 2	180 (61.2%)	84 (46.9%)	96 (53.3%)	1.9 (1.2 – 3.1)	
**Number of anti-diabetic medication**					
≤ 1	112 (38.1%)	44 (39.3%)	68 (60.7%)	Reference	0.68
≥ 2	182 (61.9%)	76 (41.8%)	106 (58.2%)	1.1 (0.7 – 1.8)	
**Insulin use**					
Yes	147 (50%)	64 (43.5%)	83 (56.5%)	1.25 (0.8 – 2.0)	0.34
No	147 (50%)	56 (38.1%)	91 (61.9%)	Reference	
**HbA1C**					
< 7	52 (17.7%)	23 (44.2%)	29 (55.8%)	Reference	0.58
≥ 7	242 (82.3%)	97 (40.1%)	145 (59.9%)	0.8 (0.5 – 1.5)	
**Medication adherence score**					
< 6	167 (56.8%)	86 (51.5%)	81 (48.5%)	Reference	< 0.01
≥ 6	127 (43.2%)	34 (26.8%)	91 (73.2%)	0.3 (0.2 – 0.6)	
**BMI**					
< 25	30 (10.2%)	7 (23.3%)	23 (76.7%)	Reference	
25 – 29	98 (33.3%)	34 (34.7%)	64 (65.3%)	1.8 (0.7 – 4.5)	0.25
≥ 30	166 (56.5%)	79 (47.6%)	84 (52.4%)	3.0 (1.2 – 7.3)	0.02

*Abbreviations*: *CI* confidence interval, *BDI-II* Beck Depression Inventory – Second edition, *BMI* Body Mass Index, *MMAS-8* Morisky Medication Adherence Scale–8 items, *OR* Odds Ratio, *HbA1C* Glycated Hemoglobin.

**Table 2:** Multivariate analysis of factors associated with depression

**Variables**		**β**	**S.E.**	**Wald**	**p-value**	**Odds ratio with 95% CI**
**Gender**	Female					Reference
	Male	-0.04	0.31	0.02	0.898	0.96 (0.52-1.77)
**Education**	Illiterate					Reference
	Elementary	-0.33	0.31	1.15	0.284	0.72 (0.39- 0.32)
	High school	-0.85	0.45	3.61	0.057	0.43 (0.18- 1.03)
	College	-1.42	0.52	7.57	0.006	0.24 (0.09-0.66)
**Occupation**	Yes					Reference
	No	1.02	0.42	6.05	0.014	2.78 (1.23-6.27)
**Additional illness**	≤ 1					Reference
	≥ 2	0.59	0.28	4.55	0.033	1.81 (1.05-3.11)
**BMI**	< 25					Reference
	25 - < 30	0.50	0.52	0.92	0.336	1.65 (0.60-4.58)
	≥ 30	0.86	0.50	3.00	0.083	2.36 (0.90-6.23)
**Medication adherence score**	< 6					Reference
	≥ 6	-1.17	0.27	14.17	0.001	0.31 (0.18- 0.53)

*Abbreviations*: *CI* confidence interval, *β* coefficient of predictor variables, *BMI* Body Mass Index, *MMAS-8* Morisky Medication Adherence Scale–8 items, *OR* Odds Ratio.

## Discussion

This study investigated the prevalence of depression among adult Palestinian type II diabetic patients and identified demographic and disease-related risk factors for depression. Our study showed that 40% of the screened patients are potential cases of depression. It should be emphasized that high BDI II scores presented in this study are not diagnostic of depression. Rather, high scores are indicative of the presence of depressive symptoms and further clinical consultation to establish definitive diagnosis of depression is needed. In our study, most patients who are potential cases of depression were females, had multiple additional illnesses, currently jobless, had low educational level, had low medication adherence, and had an abnormally high BMI. However, multivariate analysis showed that the only significant predictors of depression were low education, having no current job, having multiple additional illnesses, and having low medication adherence. Many research groups have investigated the relationship between diabetes mellitus and depression. Some research groups indicated that depression is highly prevalent among diabetics and the risk of depression might be increased in the presence of other co-morbid conditions [11,32-38]. Compared to other published studies, our results reported higher prevalence rate of depression among diabetics. A meta-analysis of 42 published studies found that the prevalence of major depression among diabetic patients was 11% and the prevalence of clinically serious depression was 31% [39]. A study in Jordan found that the prevalence rate of undiagnosed depression among Jordanian diabetic patients was 19.7% [17]. A meta-analysis study held in the United States found that the prevalence rate of depression among adult diabetic patients ranging from 3.8% to 27.3% [39]. Other studies reported a prevalence rates of 5.4% [40], 8% [41], 32.4% [42] and 41.3% [43]. One possible reason for the differences in the prevalence of depression among diabetic patients reported by different studies is the use of different scales used to screen for depressive symptoms. Some studies used the PHQ-8, others used Ham-D or BDI II scales.

The increased vulnerability to depression in individuals with type 2 diabetes is not yet clearly understood. However, depression involves physiological changes of the neuroendocrine system. The underlying cause of depression is thought to be related to changes in the neurotransmitters in the brain such as serotonin (5-HT), dopamine (DA), and norepinephrine (NE) which are monoamine neurotransmitters which affect mood and behavior. It is believed that during psychological stress counter regulatory hormones such as catecholamine a neurotransmitter, glucocorticoids, growth hormones, and glucagons are activated [44]. The activation of the counter regulatory hormones interferes in the action of insulin which is not able to lower glucose but instead elevates blood glucose. The increase in glucose level creates a greater challenge in maintaining metabolic control. Poor glycemic control and functional impairment due to increasing diabetes complications may cause or worsen depression and lessen the response to antidepressant treatment [45]. In spite of the known devastating effect of depression on diabetes, it was found that only 31% of patients with diabetes and depression received adequate antidepressant treatment and only 6% received 4-5 sessions of psychotherapy in a 12 month period [46]. Studies of the economics of treatment of depression among diabetic patients have yielded positive results. The health care expenses sustained by individuals with diabetes and depression are higher than those with diabetes alone [47,48].

It may be argued that the high prevalence rate of depression found in our study is due to many patients having uncontrolled diabetes (82%). However, we did not find significant relationship between glycemic control and potential for depression. Studies about the relationship between depression and poor glycemic control gave mixed results. A study had shown a negative relationship between depression and poor glycemic control and diabetes complications. Worse glycemic control was observed in depressed adults with diabetes [49]. A meta-analysis study [50] found that patients with type 1 and type 2 diabetes and depression persistently had higher HbA1c levels [50]. Wagner et al. also found higher HbA1c and more diabetes complications in African Americans with higher depressive symptoms after controlling for confounders between depression and HbA1c levels [51]. In a prospective representative study of patients with type 2 diabetes, depression predicted problems with medication adherence, and unsatisfactory glycemic control [52]. Diabetic complications and mortality were also found to be greater among depressed patients [14,53,54]. The microvascular and macrovascular complications of diabetes are augmented by the presence of depression in diabetes thus contributing to the increased mortality rate in this population [55]. In contrast, other investigators found no relationship between depression and diabetic complications [42,56-58]. Cross-sectional studies found a significant positive correlation between depressive symptoms and HbA1c in patients with Type 1 diabetes but no significant correlation in patients with Type 2 diabetes [59-61], indicating that depression affects glycemic control in patients with Type 1 but not Type 2 diabetes. In support of this, a study found that patients with Type 1 but not Type 2 diabetes who had depression showed significantly worse glycemic control than their counterparts who had no depression [62,63]. A study showed that changes in depressive symptoms were not associated with changes in HbA1c or fasting glucose levels over a 1-year period in either patients with Type 1 or Type 2 diabetes [63].

Our study showed that approximately two thirds of those who scored ≥ 16 points were females. The higher prevalence of depression among females is in consistence with other studies [17,35,55,64,65]. In a meta-analysis, Anderson et al found that diabetes doubles the risk of depression and it is especially more among females [39]. However, not all studies reported this gender differences in depression [58]. Our results indicated that about 90% of the total sample was either overweight or obese (had a BMI > 25). The BDI score was significantly higher among obese patients. A study found that depression was more common among diabetic women especially if they were overweight and that body weight was a predictor of depression more than diabetes itself [66]. Similarly, recent studies have found that higher BMI was a predictor of depression in type 2diabetes [64,67]. High educational level decreases the odds of being classified as depressed patients which is similar to findings published in other studies [13,17]. Medication adherence showed a significant relationship with depression. Adherent patients are associated with lower odds of being depressed. It has been reported by other researchers that depressed diabetic patients do not pay much effort on daily management activities [68,69] and this result is consistent with many studies that reported that depressed diabetic patients are likely to have physical limitations and a poor quality of life [70-73], bearing in mind that self-care behaviors in diabetes include adherence to dietary restrictions and medications, adequate physical exercise and blood glucose monitoring [68,69]. Management guidelines for diabetes mellitus emphasize the importance of medication adherence, physical activity, diet and self-monitoring of blood glucose [74]. Gonzalez et al. proposed that the presence of depressive symptoms are good predictors of poor adherence to self-care particularly in adherence to medications and diet and exercise regimens [75]. A systematic review of treatment adherence among individuals with diabetes and depression indicated that there was a significant relationship between depression and treatment non-adherence [75]. Finally, our study indicated that having no work is significantly associated with depression score ≥ 16 points. This is expected since having no work is by itself a depressing factor.

In contrast to some other studies, our study showed no significant association between depression score and age among diabetic patients [76]. Furthermore, our study showed no significant association between depression and the use of insulin which is contrary to other published studies [11]. A study reported that delayed initiation of insulin in type 2 makes a significant number of diabetic patients vulnerable to diabetic complications and its adverse outcomes, including depression [77]. Our study found that the duration of DM was not significantly associated with depression which is consistent with the findings reported by other studies [78,79]. However, because the incidence of diabetic complication increases with increased illness duration, one could expect greater depression risk in those who have been ill for a longer time. Some research groups reported a significantly higher rate of depression in individual with long standing history of diabetes than in the newly diagnosed diabetic [80].

Our study is the first to be conducted in Palestine to determine the prevalence of undiagnosed depression among diabetic patients. However, our study has few limitations. First, this study is cross-sectional where causal relationship between diabetes and depression cannot be established. Variables identified as significantly associated with depression may precede depression, but in some cases, these variables could also occur as a result of depression; thus, high scores of BDI II among diabetic patients must be interpreted with caution. Further studies with longitudinal prospective design and the presence of age and gender matched group is needed to shed more light on the potential relationship between depression and diabetes mellitus. It is important to state that depression screening measures provide an estimation of the severity of depressive symptoms and assess the severity within a specific period of time but they do not diagnose depression. However, those who score high in depression measure scales need to interviewed and assessed for a confirmation of depression. The second limitation of this study was its partial reliance on self-report for its measures, including depression and medication adherence; therefore, a clinical interview to assess depression maybe a superior measure because of its higher level of specificity. Accordingly, a longitudinal clinical study of a community-based sample using clinical symptoms for diagnosis of depression is needed to assess the relationship between various variables including diabetes control. Third limitation of this study was HbA1c measurement. The presence of many clinically silent hemoglobin variants might cause deviation in the HbA1c results leading to falsely high or low values [81,82]. Therefore, the accuracy of the results and validity of the HbA1c interpretation cannot be assured. Finally, gender differences in risk of depression among diabetic patients need further investigation using large sample size is needed since univariate analysis showed such gender differences with higher risk among females. Community-based study among diabetic women will be of great value given that women are more prone to depression than men.

## Conclusion

The prevalence of depression among Palestinian diabetic patients is higher than reported in other communities and has never been approached before. Being a female, not adherent to anti-diabetic medications, having low educational level and being jobless were significant predictors and are associated with an increased likelihood of developing major depressive disorders. We highly recommend the introduction of the psychological aspect among the diabetic health care plan to reduce the number of the depressed or the misrecognized depressed diabetic patients and consequently offer them a better quality of life.

## Abbreviations

BDI-II: Beck depression inventory – second edition; HbA1C: Glycated hemoglobin; MMAS-8: Morisky medication adherence scale – 8 items; IRB: Institutional review board; BMI: Body mass index; DM: Diabetes mellitus.

## Competing interests

The authors declare that they have no competing interests.

## Authors’ contributions

All authors were involved in drafting the article and all authors approved the final version to be submitted for publication. All authors have added an intellectual significant value to the manuscript. H.A was involved in subject recruitment and interview, data collection, data coding and entry, literature review, and manuscript editing. This was done in partial fulfillment of a master degree in Community Mental Health Nursing at An-Najah National University. S.A and S.Z were involved in concept, study design, manuscript writing and editing, and academic co-supervision for H.A according to An-Najah university regulations. W.S was involved in the study conception and design, literature review, manuscript writing, manuscript submission, manuscript revision, head of the research group, and academic supervision for H.A according to An-Najah university regulations. This manuscript is part of a project for master degree in the graduate program of Community Mental Health Nursing. The project was initially and originally conceptualized and designed by the Clinical Pharmacology/ Toxicology Research Group at An-Najah National University (W.S, S.Z, and S.A). The project was then assigned to H.A as a thesis project and was academically supervised by W.S and S.A in adherence to An-Najah University regulations with regard to academic supervision for graduate students.

## Authors’ information

Professor Waleed M. Sweileh is the head of a research group (S.Z, S.A, A.S and A.A) which has published in the field of clinical pharmacology, toxicology, pharmacoepidemiology, social and community pharmacy, clinical pharmacy and medicine. The research group has also supervised many students in the fields of nursing, public health and pharmacy.

## Pre-publication history

The pre-publication history for this paper can be accessed here:

http://www.biomedcentral.com/1471-2458/14/163/prepub
